# Refractory urticarial dermatitis successfully treated with mycophenolate mofetil

**DOI:** 10.1093/omcr/omag140

**Published:** 2026-07-27

**Authors:** Hugo J Leme, Marcelo Pacheco Silva, Adelina Costin, Ana Isabel Gouveia, João Alves

**Affiliations:** Dermatology Department, Unidade Local de Saúde de Almada-Seixal, Av. Torrado da Silva, 2805-267, Almada, Portugal; Dermatology Department, Unidade Local de Saúde de Almada-Seixal, Av. Torrado da Silva, 2805-267, Almada, Portugal; Dermatology Department, Unidade Local de Saúde de Almada-Seixal, Av. Torrado da Silva, 2805-267, Almada, Portugal; Dermatology Department, Unidade Local de Saúde de Almada-Seixal, Av. Torrado da Silva, 2805-267, Almada, Portugal; Dermatology Department, Unidade Local de Saúde de Almada-Seixal, Av. Torrado da Silva, 2805-267, Almada, Portugal

**Keywords:** dermatology, pharmacology and pharmacy, urticarial dermatitis, mycophenolate mofetil, refractory dermatosis

## Abstract

Urticarial dermatitis is an uncommon inflammatory dermatosis characterized by persistent pruritic urticarial and eczematous eruptions associated with a dermal hypersensitivity reaction pattern on histopathology. We report a case of severe refractory urticarial dermatitis resistant to multiple therapies, including corticosteroids, cyclosporine, methotrexate, dapsone and azathioprine, which demonstrated significant clinical improvement following treatment with mycophenolate mofetil. This case highlights the diagnostic challenges of urticarial dermatitis and supports mycophenolate mofetil as a potential therapeutic option in refractory disease.

## Introduction

Urticarial dermatitis is an uncommon inflammatory dermatosis characterized by persistent pruritic urticarial and eczematous lesions with histopathological features of dermal hypersensitivity [1]. The condition may significantly impair quality of life and is frequently underrecognized due to its overlapping clinical and histological features with other inflammatory dermatoses [2]. We report a case of refractory urticarial dermatitis successfully treated with mycophenolate mofetil.

## Case report

A 78-year-old woman with no previous medical history presented with a 12-year history of a pruritic dermatosis. She underwent several skin biopsies compatible with drug eruption. However, the patient denied having taken any new medications. She was initially treated with oral antihistamines, topical corticosteroids, several intermittent cycles of oral corticosteroids, cyclosporine 100 to 200 mg daily, methotrexate 7.5 mg weekly, dapsone 50 to 100 mg daily and azathioprine 100 mg daily. However, these therapies failed to achieve significant clinical improvement, and the disease followed a chronic relapsing course.

**Figure 1:**
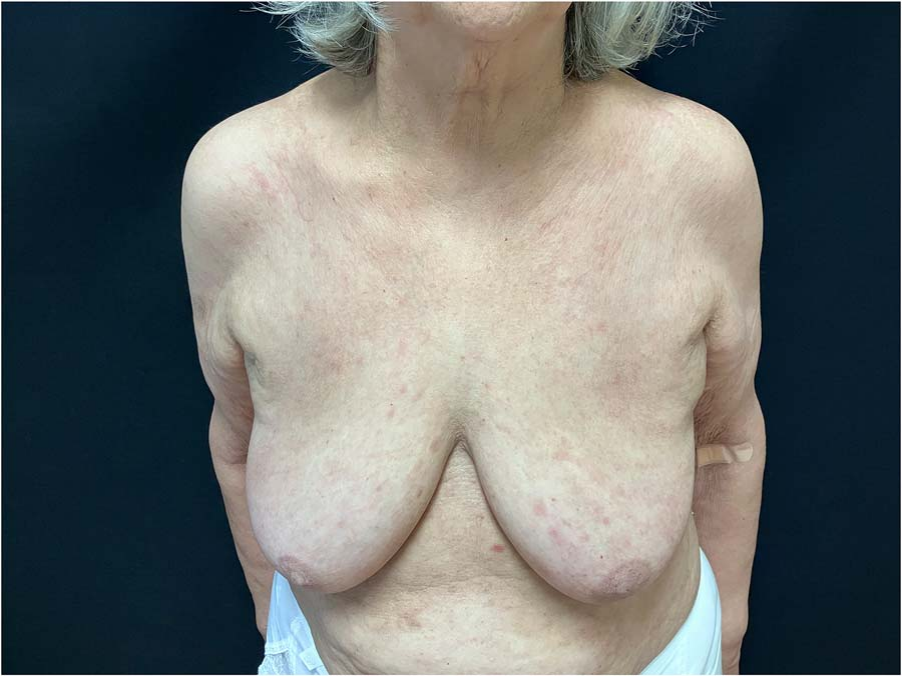
Erythematous urticarial-like papules and plaques admixed with eczematous lesions involving the trunk.

On dermatological examination, the patient had erythematous urticarial-like papules and plaques admixed with eczematous lesions involving the trunk, back and lower limbs (Fig. 1).

**Figure 2:**
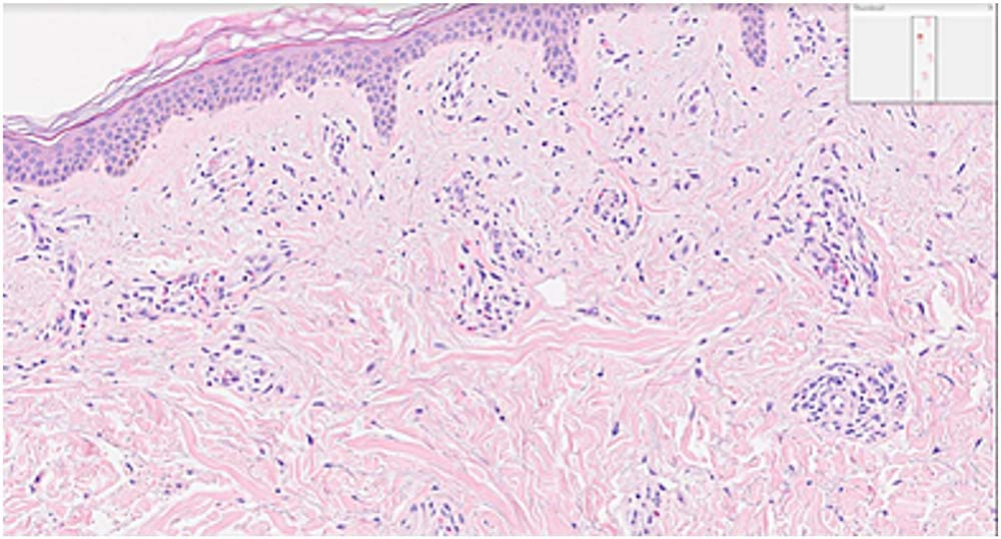
Moderate superficial perivascular inflammatory infiltrate composed of lymphocytes, histiocytes and eosinophils, with mild papillary dermal oedema and no evidence of vasculitis.

After clinicopathological correlation and literature review, a repeat skin biopsy revealed a moderate superficial perivascular inflammatory infiltrate composed of lymphocytes, histiocytes and eosinophils, associated with mild papillary dermal oedema and no evidence of vasculitis (Fig. 2).

A diagnosis of urticarial dermatitis was made. Mycophenolate mofetil was initiated at 1 g twice daily (2 g/day) for five months, followed by tapering to 1.5 g/day for two weeks and subsequently 500 mg twice daily for one month, with marked improvement of the dermatosis and pruritus and no recurrence during a 9-month follow-up period.

Age-appropriate malignancy screening was performed and yielded negative results.

## Discussion

Urticarial dermatitis was described in 2006 as a histopathological skin reaction pattern with a variable clinical spectrum [1, 3]. The clinical features of urticarial dermatitis represent a reaction pattern that may be observed in a number of dermatological conditions, including various subtypes of dermatitis, drug eruption, bullous pemphigoid and scabies [4]. Nevertheless, Kossard and colleagues have demonstrated that in a subset of patients, urticarial dermatitis may represent a distinct clinicopathological entity that cannot be further classified [5].

The histological features of urticarial dermatitis usually include a normal stratum corneum, minimal spongiosis and upper dermal perivascular and interstitial lymphocytic infiltrate, with a variable amount of eosinophils [5]. Although these findings are not entirely specific and may overlap with other eczematous dermatoses, the combination of papillary dermal oedema, eosinophil-rich superficial infiltrate and the characteristic chronic urticarial-eczematous eruption supported the diagnosis after clinicopathological correlation.

Due to the paucity of large-scale studies examining this condition, the optimal initial workup for these patients remains unclear [4]. Some studies have shown an association between urticarial dermatitis and systemic malignancy, which reveals the importance of screening for internal malignancies, especially in recalcitrant cases [6]. Other proposed triggers and associations include medications, chronic eczematous dermatoses, autoimmune diseases, and infections. In many patients, however, no specific trigger can be identified despite extensive investigation [4].

The most commonly used treatments for urticarial dermatitis are oral antihistamines, topical and systemic corticosteroids, narrowband ultraviolet B radiation and topical calcineurin inhibitors [1]. In addition to these therapies, there are some reports involving dapsone, mycophenolate mofetil and cyclosporine [2]. Chaptini and Sidhu showed two cases of severe urticarial dermatitis that were refractory to standard treatment and improved with mycophenolate mofetil [5]. Despite the widespread use of mycophenolate mofetil in Dermatology, there is a paucity of studies and evidence supporting its use in urticarial dermatitis [5, 7].

Data regarding biologic therapies in urticarial dermatitis are extremely limited. Nevertheless, dupilumab may represent a potential therapeutic option given its efficacy in chronic pruritic and eczematous disorders and its emerging role in chronic spontaneous urticaria [8]. Likewise, omalizumab could theoretically benefit patients with prominent urticarial features based on its established efficacy in chronic spontaneous urticaria [9]. However, evidence supporting the use of either biologic in urticarial dermatitis is currently lacking, and further studies are required to clarify their role.

In this case, the favourable response to mycophenolate mofetil may be explained by its selective inhibition of inosine monophosphate dehydrogenase, resulting in suppression of T- and B-lymphocyte proliferation and attenuation of cytokine-mediated inflammation [7]. Given the presumed immune-mediated pathogenesis of urticarial dermatitis and the superficial lymphoeosinophilic infiltrate and papillary dermal oedema observed on histopathology, mycophenolate mofetil may have provided a broader and more sustained immunomodulatory effect than previously administered therapies, ultimately leading to disease control. Further studies are needed to better define its role in refractory urticarial dermatitis.

This case highlights the diagnostic and therapeutic challenges associated with urticarial dermatitis, emphasizing its importance as a differential diagnosis in patients presenting with chronic urticarial and/or eczematous dermatoses. It further supports mycophenolate mofetil as a potential therapeutic option in severe refractory disease.
